# Synergistic Induction of Interferon α through TLR-3 and TLR-9 Agonists Identifies CD21 as Interferon α Receptor for the B Cell Response

**DOI:** 10.1371/journal.ppat.1003233

**Published:** 2013-03-14

**Authors:** Dhohyung Kim, Stefan Niewiesk

**Affiliations:** Department of Veterinary Biosciences, Ohio State University, Columbus, Ohio, United States of America; University of North Carolina at Chapel Hill, United States of America

## Abstract

Maternal antibodies inhibit seroconversion and the generation of measles virus (MeV)-specific antibodies (both neutralizing and non-neutralizing antibodies) after vaccination whereas T cell responses are usually unaffected. The lack of seroconversion leaves individuals susceptible to vaccine-preventable infections. Inhibition of antibody secretion is due to the inhibition of B cells through a cross-link of the B cell receptor with the inhibitory FcγIIB receptor (CD32) by maternal antibody/vaccine complexes. Here, we demonstrate that a combination of TLR-3 and TLR-9 agonists induces synergistically higher levels of type I interferon in vitro and in vivo than either agonist alone. The synergistic action of TLR-3 and TLR-9 agonists is based on a feedback loop through the interferon receptor. Finally, we have identified CD21 as a potential receptor for interferon α on B cells which contributes to interferon α-mediated activation of B cells in the presence of maternal antibodies. The combination leads to complete restoration of B cell and antibody responses after immunization in the presence of inhibitory MeV-specific IgG. The strong stimulatory action of type I interferon is due to the fact that type I interferon uses not only the interferon receptor but also CD21 as a functional receptor for B cell activation.

## Author Summary

Maternal antibodies provide protection against infection with pathogens early in life but also interfere with vaccination. This interference is caused by a vaccine/maternal antibody complex which links the B cell receptor to the inhibitory CD32 molecule. Here, we show that this cross-link results in impaired B cell activation and proliferation which is correlated with diminished antibody responses. We also found that induction of large amounts of type I interferon restores the neutralizing antibody response in the presence of maternal antibodies. The best induction of type I interferon was accomplished by a combination of known activators of interferon secretion (a combination of TLR-3 and TLR-9 agonists). The strong stimulation by interferon is due to the previously unappreciated role of CD21 as functional receptor for interferon alpha. Our findings demonstrate that the dual receptor usage of type I interferon receptor and CD21 is crucial for B cell activation in the presence of maternal antibodies. This study suggests that measles vaccine, and potentially other vaccines, may induce optimal antibody responses when they are reconstituted with TLR-3 and TLR-9 agonists and thus these agonists may have great potential for clinical use.

## Introduction

A fundamental unresolved issue in vaccinology is the inhibition of vaccination against infectious diseases of humans [1], [2],[3], [4], [5], [6], [7] and animals [8], [9], [10], [11], [12], [13], [14], [15], [16], [17] by maternal antibodies. Studies in patients as well as experiments in animal models testing adjuvants and vaccine vectors have shown that maternal antibodies do not inhibit T cell responses [18], [19], [20], [14]. However, if protection (at least partially) depends on the B cell response and production of neutralizing antibodies (as it does for measles virus and many other pathogens), vaccination regularly fails.

Worldwide, close to 200,000 children die of measles virus every year. During their first year of life, children are protected by neutralizing maternal antibodies against MeV infection. Over time, these antibody titers wane and eventually do not protect against wildtype virus infection (for review [21]). However, even these low non-protective antibody titers inhibit the generation of MeV-specific antibodies (both neutralizing and non-neutralizing antibodies) but not the development of a MeV-specific T cell response [18]. As neutralizing antibodies but not T cells protect against infection [19], [22], [23], these children are susceptible to MeV infection. We have used the cotton rat (Sigmodon hispidus) model of measles vaccination to analyze the inhibitory mechanism of maternal antibodies because the cotton rat is the only rodent in which measles virus after intranasal inoculation replicates in the respiratory tract and lymphoid organs [24]. In this animal model, we have been able to demonstrate that both natural maternal MeV-specific IgG antibodies, as well as passively transferred human and mouse MeV-specific IgG are able to inhibit the generation of MeV-specific antibodies (both neutralizing and non-neutralizing antibodies) after immunization [19], [25], [26]. B cell inhibition is due to cross-linking of the B cell receptors (BCR) and Fcγ receptors IIB (FcγRIIB) by a complex formed by maternal IgG and the MeV vaccine [26]. This inhibitory effect can be partially overcome by activation of B cells through cross-linking BCR and complement receptor 2 (CR-2/CD21) with a complex of MeV vaccine, MeV-specific IgM and complement protein C3d [26].

Two viral vector systems (vesicular stomatitis virus (VSV) and Newcastle Disease virus (NDV)) which express measles virus hemagglutinin (H) can induce H specific neutralizing antibodies after vaccination in the presence of inhibitory MeV-specific IgG. In contrast to measles virus, both VSV [27] as well as NDV induce type I interferon [28]. For NDV we have shown that its ability to induce neutralizing antibodies correlates with its ability to induce type I interferon in cotton rat plasmacytoid dendritic cells, and in cotton rat lung tissue [28]. In vitro, neutralization of IFN α abrogates stimulation of B cell responses by NDV. Viruses induce type I interferon through viral nucleic acids which are recognized by TLR-3 (single-stranded RNA), TLR-7 (double-stranded RNA) and TLR-9 (DNA). We used a combination of TLR-3 and TLR-9 agonists in order to mimic infection with RNA and DNA viruses, to increase the induction of type I interferon and fully restore the B cell response after vaccination in the presence of inhibitory MeV-specific IgG. TLR-3 signals through the TRIF/IRF-3 pathway and mainly induces IFNβ whereas TLR-9 signals through the MyD88/IRF-7 pathway and mainly induces IFNα. It was hypothesized that the combination of these agonists would result in superior induction of type I interferon because of synergistic effects mediated through the interferon receptor feedback loop. We were able to demonstrate higher induction of type I interferon, and a complete restoration of the B cell response after immunization with MeV and a TLR-3/TLR-9 agonist combination in the presence of inhibitory MeV-specific IgG. In addition, it was shown that IFNα uses CD21 as a functional receptor (in addition to IFN receptor) to drive B cell responses, and this dual receptor usage likely explains its strong stimulatory potential for the B cell inhibited by maternal antibodies.

## Results

### Synergistic type I interferon induction by a combination of TLR-3 and TLR-9 agonists in vitro and in vivo

**Figure 1:**
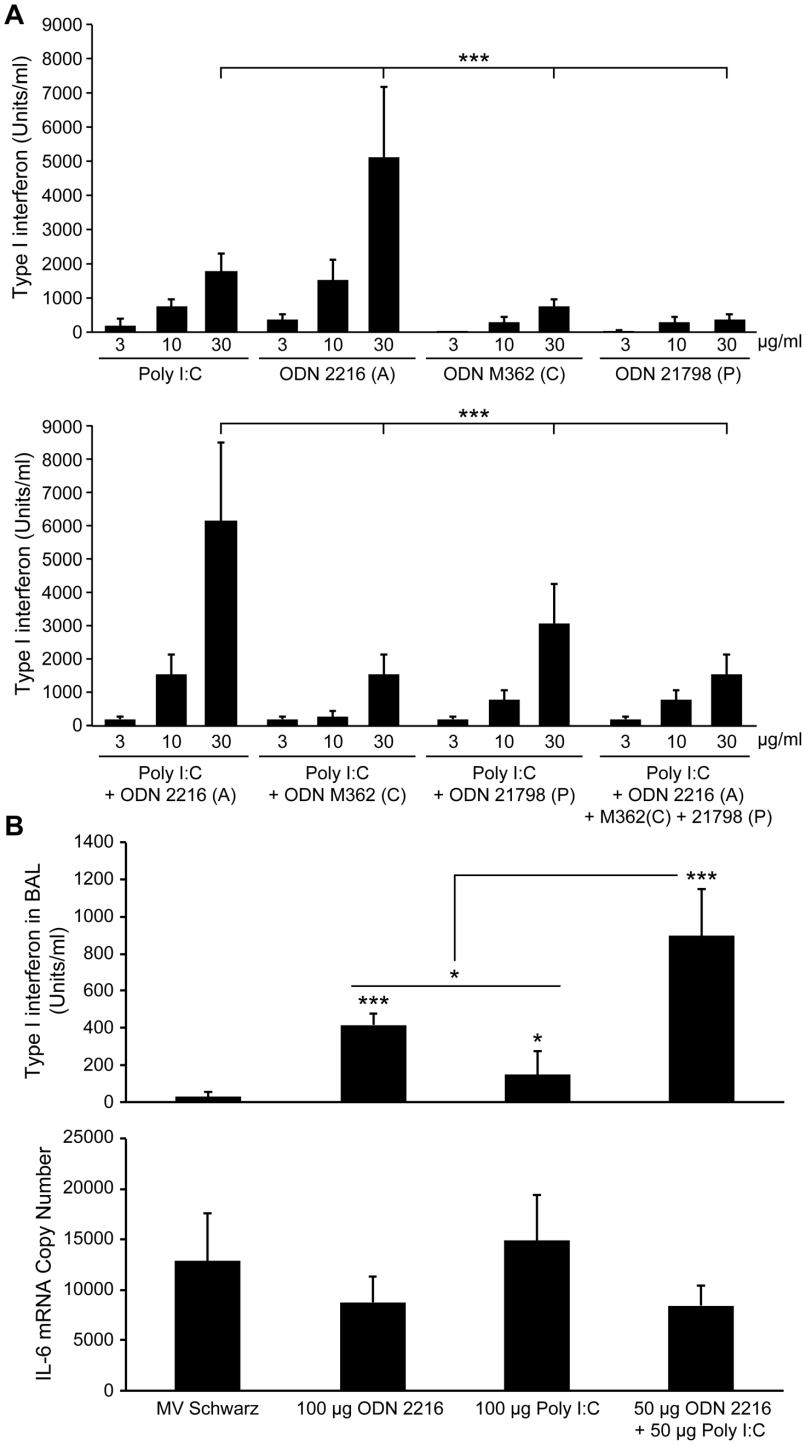
Synergistic effect of ODN 2216 and poly I:C on type I interferon induction in vitro and in vivo. (A) Bone-marrow derived cotton rat plasmacytoid dendritic cells were cultured with oligonucleotides for 24 hours, and type I interferon was measured from culture supernatant by bioassay. ODN 2216 (type A), M362 (type C) ODN 21789 (type P) and poly I:C were tested alone (upper panel) or in combination (lower panel). The amount of the combination was equal to the amount of single agonist. Results are combined from three independent experiments. The difference between 30 ug of poly I:C and M362 (type C) or ODN 21789 (type P) was significant, too (p<0.05). (B) The induction of type I interferon in the lungs of cotton rats was measured after intranasal inoculation of either 10^5^ pfu of MeV vaccine (Schwarz strain) or 100 µg of oligonucleotide. At 24 hours post inoculation, bronchioalveolar lavage (BAL) fluid was collected and tested for the presence of type I interferon by bioassay. BAL cell pellets were used to quantify the level of cotton rat IL-6 by quantitative PCR. The results reflect four animals per group ± SD, and are representative of three experiments. One unit of type I interferon equals one picogram. Statistical analysis was done by ANOVA (* p<0.05, *** p<0.001).

We have shown previously that two viral vector systems which express measles virus hemagglutinin can induce H specific neutralizing antibodies after vaccination in the presence of inhibitory MeV-specific IgG. NDV is able to induce type I interferon in cotton rat plasmacytoid dendritic cells, and in cotton rat lung tissue. For VSV, we tested its ability to induce type I interferon in cotton rat plasmacytoid dendritic cells and found levels of 128 U/10^6^ pDC (moi of 1). These results led us to try to improve the stimulation of the B cell response by using TLR-3 and TLR-9 agonists as inducers of type I interferon. We tested pI:C as TLR-3 agonist and human ODN type A, C and P as TLR-9 agonists on plasmacytoid dendritic cells individually and in combination. The combination of pI:C and human ODN 2216 (type A) proved to induce the highest levels of type I interferon in plasmacytoid dendritic cells (Figure 1A). In lung tissue, the combination of pI:C and ODN 2216 was also able to induce higher amounts of type I interferon then either agonist alone (Figure 1B). It is worth noting that the combination of pI:C and ODN 2216 induced higher levels of type I interferon than either NDV (256 U/ml [28]) or VSV (128 U/mL) in plasmacytoid dendritic cells, or NDV in cotton rat lung tissue (250 U/ml [28]). The second cytokine to influence the development of human and mouse plasma cells is IL-6. Similar to these species, cotton rat IL-6 stimulates B cells in an ELISPOT assay (data not shown). However, pI:C and ODN 2216 did not induce higher levels of IL-6 in broncho-alveolar lavage cells than measles virus alone (Figure 1B).

**Figure 2:**
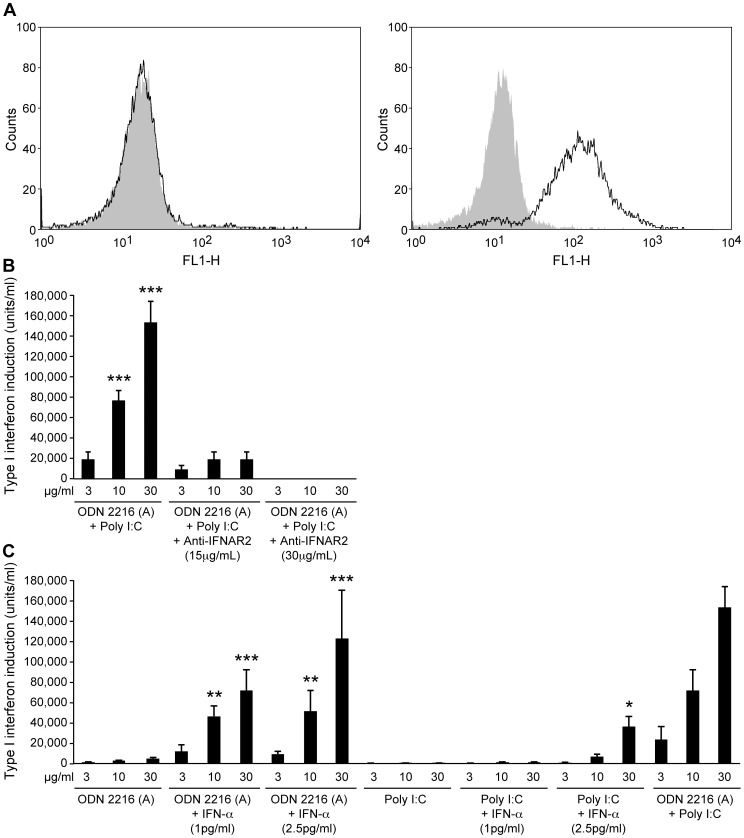
Synergistic effect of ODN 2216 and poly I:C depends on positive feedback regulation of type I interferon induction via the interferon receptor. (A) Spleen cells from cotton rats were stimulated with Concanavalin A for 72 hours and lymphoblasts purified by centrifugation on a ficoll-gradient. Subsequently, cells were stained with antisera specific for the human interferon receptor to test for crossreactive binding to cotton rat cells and analyzed by flow cytometry. Antibody specific for human interferon alpha receptor-1 (IFNAR-1) did not react with cotton rat cells (left) whereas antibody specific for human interferon alpha receptor-2 (IFNAR-2) did (right). IFNR specific antibody is shown as black line, controls as gray areas. B. IFNAR-2 antibody was added to cotton rat spleen cells. One hour later different concentrations of a combination of ODN2216 and poly I:C was added. 24 hours later supernatant was harvested and tested for the presence of type I interferon. The data is combined from two independent experiments. Significant differences were seen between cells treated with the combination of ODN2216 and poly I:C alone, or the combination of ODN2216 and poly I:C with antibody. IFNAR-1 antibody (not reactive with cotton rat cells) had no effect. C. Cotton rat spleen cells were stimulated with ODN 2216 or poly I:C, with or without the addition of recombinant cotton rat interferon alpha. (One unit equals one picogram of type I interferon.) 24 hours later supernatant was harvested and tested for the presence of type I interferon. The data is combined from two independent experiments, and significance was compared to cells without type I interferon treatment. Statistical analysis was done by ANOVA (* p<0.05, ** p<0.01, *** p<0.001).

In order to test whether the action of the TLR agonist combination acts through the IFN receptor (IFNR) feedback loop, we blocked the signaling chain of the IFNR with antibody (Figure 2A and B). This inhibited the type I interferon response in an antibody concentration dependent manner. In a reverse experiment, we demonstrated that even minute amounts of interferon alpha in combination with either pI:C or ODN 2216 were able to significantly improve the induction of type I interferon induced by either pI:C or ODN 2216 alone, again emphasizing the necessity for the IFNR feedback loop (Figure 2C).

### Stimulation of B cell responses through combination of TLR-3 and TLR-9 agonists

**Figure 3:**
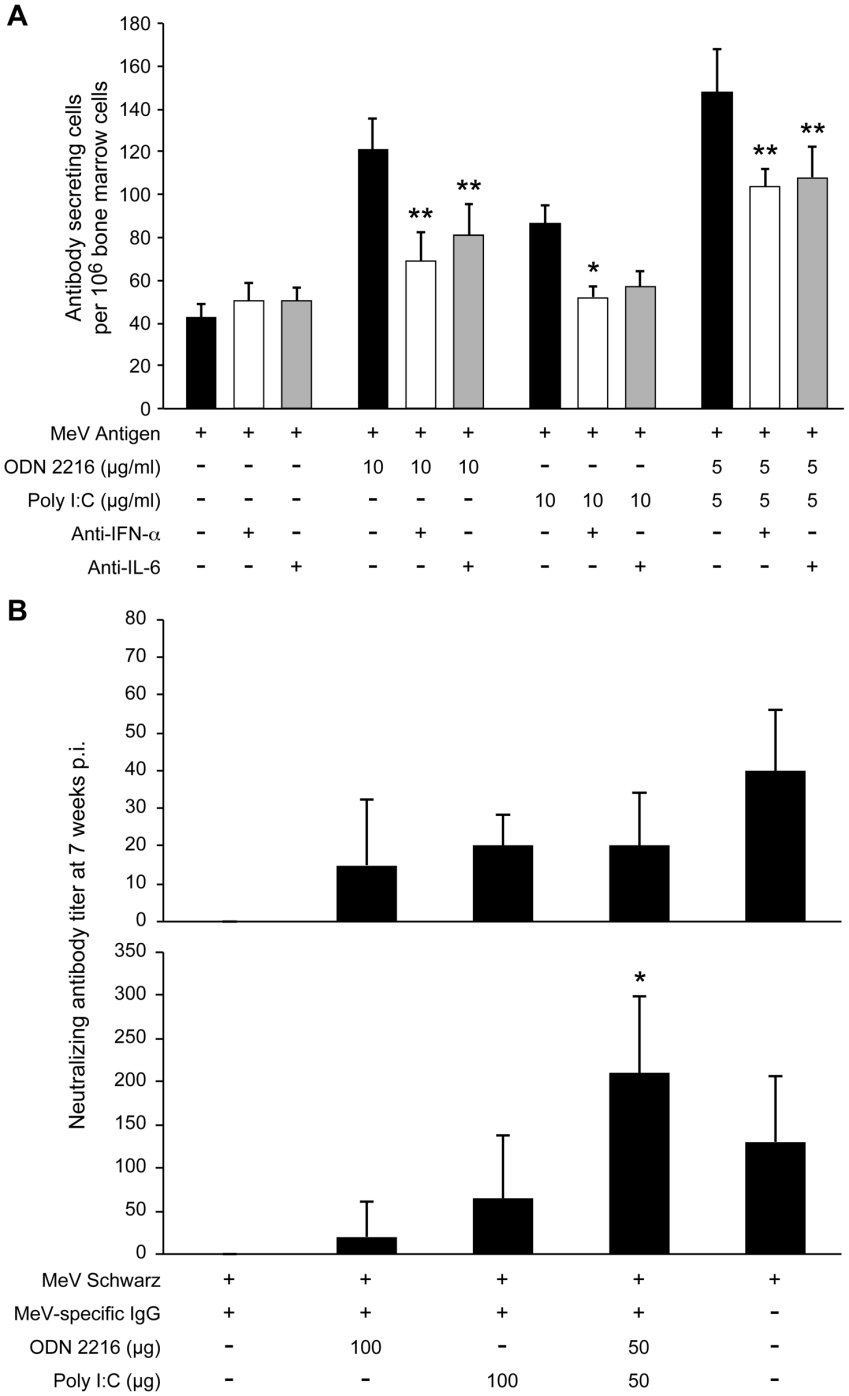
ODN 2216 and poly I:C stimulate B cell activation in vitro and restore the neutralizing antibody response after immunization in the presence of MeV-specific IgG. A) The number of MeV-specific B cells was measured from bone marrow cells of MeV-immune cotton rats. The addition of ODN 2216 and poly I:C individually and in combination increased B cell numbers whereas the addition of sera neutralizing cotton rat interferon alpha and IL-6 reduced this stimulation. Each bar graph represents the mean ± SD of triplicate wells. B) Cotton rats were immunized intranasally (upper panel) or subcutaneously (lower panel) with MeV, or MeV with ODN 2216 and/or pI:C in the presence or absence of human MeV-specific IgG (neutralization titer of 100) which had been injected intraperitoneally one day before immunization. Sera were collected at seven weeks post vaccination and the titer of neutralizing antibody was determined by neutralization assay. Each bar graph represents the average titer of four animals ± SD. The experiment is representative of three experiments. Statistical analysis was done by ANOVA (* p<0.05, ** p<0.01, *** p<0.001).

In order to test the ability of the pI:C/ODN2216 combination to stimulate B cell and antibody responses, the TLR agonists were used in an ELISPOT assay. Both pI:C and ODN 2216 stimulated higher B cell responses from the bone marrow of MeV-immune cotton rats but the combination was superior to either agonist alone (Figure 3A). Stimulation of B cells by TLR agonists could be reduced with neutralizing antibody against type I interferon as well as IL-6. This is consistent with the role of sequential action of type I interferon and IL-6 for B cells to transition from activated B cells to plasmablasts (type I interferon), and then from plasmablasts to antibody secreting plasma cell (IL-6) [29].

When cotton rats are immunized with MeV either intranasally or subcutaneously, the generation of neutralizing antibodies is suppressed by inhibitory MeV-specific IgG. If MeV was supplemented with either pI:C, ODN 2216, or a combination of both, the induction of neutralizing antibodies was detected (Figure 3B). After i.n. immunization (Figure 3B, upper panel) both agonists alone and the combination induced comparable levels of neutralizing antibody levels. However, these titers were lower than after immunization with MeV in the absence of inhibitory antibody. After s.c. immunization (Figure 3B, lower panel), either agonist alone induced neutralizing antibodies, but the combination of pI:C/ODN 2216 induced higher antibody levels than either agonist alone, and antibody levels were comparable to MeV immunization in the absence of inhibitory MeV-specific IgG.

### MeV-specific inhibitory IgG inhibit B cell development

**Figure 4:**
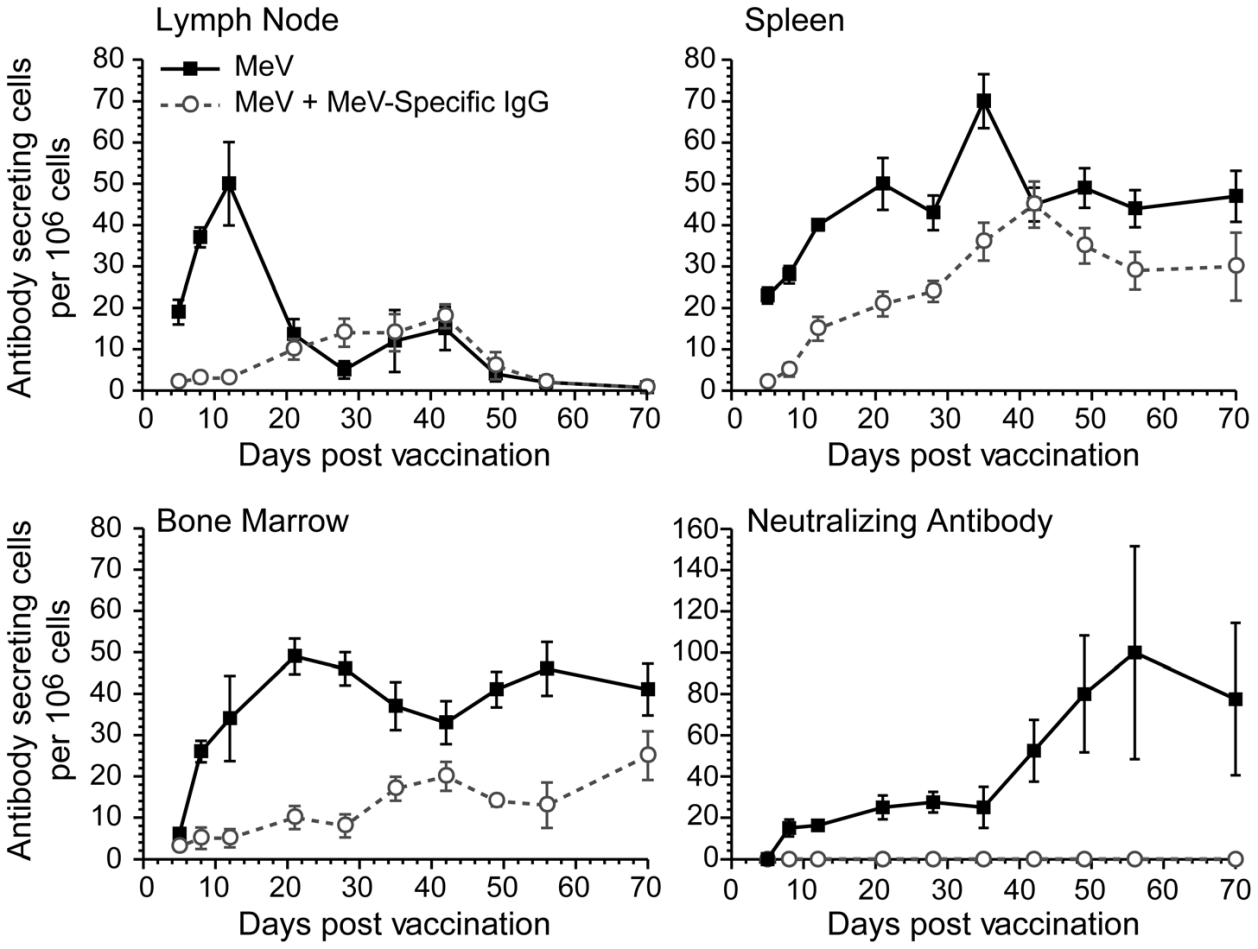
Kinetic of B cell and antibody after immunization in the absence and presence of MeV-specific IgG. Cotton rats were immunized intranasally with 10^5^ pfu MeV in the absence (solid line) or presence of MeV-specific IgG (NT of 100, dotted line). The number of MeV-specific B cells was measured in the lung draining mediastinal lymph nodes, spleen and bone marrow at indicated time points post vaccination by B cell ELISPOT.

After immunization in the presence of maternal antibodies, the generation of neutralizing antibodies is inhibited. This could be due to a lack of B cell generation, or a lack of antibody secretion by B cells. In order to determine B cell development after immunization with MeV, cotton rats were immunized i.n., and mediastinal lymph nodes (MDLN; which drain the lung), spleen and bone marrow were tested for the presence of MeV-specific B cells on day 5, 8, 12 and in weekly intervals between week 3 and 10 by ELISPOT (Figure 4). Antibody levels in serum were determined by neutralization assay. After MeV immunization in the presence of MeV-specific IgG, no neutralizing antibodies were produced (NT below the threshold of 10), and the level of total MeV-specific antibodies was low (maximal average 1∶500 by endpoint titration). Immunization in the absence of MeV-specific IgG led to measurable neutralizing antibodies from day 8 and to increased total MeV-specific antibody titers (maximal average 1∶5000 by endpoint titration). In MDLN from animals immunized in the absence of MeV-specific IgG, numbers of MeV-specific B cells peaked at day 12 and declined towards day 21. B cell numbers in spleen and bone marrow increased steadily over time and remained stable for at least ten weeks. After immunization in the presence of MeV-specific antibodies, B cells in MDLN proliferated with a delay of three weeks at low levels. MeV-specific B cells accumulated in bone marrow slower and to lower numbers than in animals immunized in the absence of MeV-specific IgG. The interesting result was the accumulation of relatively high numbers of B cells in the spleen without generation of neutralizing antibodies. These data indicate that the early activation and proliferation of B cells in MDLN is necessary for subsequent population of spleen and bone marrow, and is blunted in animals immunized in the presence of MeV-specific IgG. The lack of stimulation in the early phase of the B cell response correlates with low long term immune responses (no neutralizing antibodies and low B cell numbers in bone marrow).

### Combination of TLR-3 and TLR-9 agonists restores B cell response after immunization in the presence of MeV-specific inhibitory IgG

**Figure 5:**
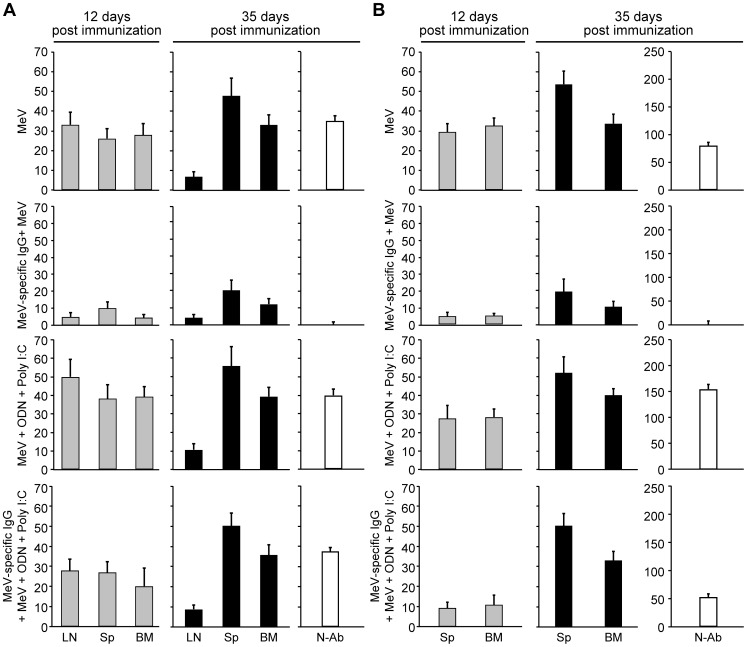
Kinetic of B cell generation is restored to normal levels in the presence of MeV-specific IgG after co-immunization with ODN 2216 and poly I:C. Groups of cotton rats were immunized with 10^5^ pfu MeV vaccine alone or with the combination of ODN2216 (50 µg) and poly I:C (50 µg) in the absence or presence of MeV-specific IgG. At 12 days and 35 days post vaccination, generation of MeV-specific B cells in the lung draining mediastinal lymph nodes (LN), spleen (Sp) and bone marrow (BM) and induction of neutralizing antibodies (N-Ab) after immunization by the intranasal route (A) or by the subcutaneous route (B) were measured by B cell ELISPOT and neutralization assay. Each bar graph represents the average of four animals ± SD.

In order to test the stimulatory effect of the pI:C/ODN 2216 combination on MeV immunization, one set of cotton rats was immunized i.n. and B cell numbers in lymph nodes, spleen and bone marrow were measured on day 12 and day 35 after immunization (Figure 5A). A second set of cotton rats was immunized s.c. and B cell numbers in spleen and bone marrow were measured on day 12 and day 35 after immunization (Figure 5B). In addition, sera were tested for neutralizing antibodies at day 35. After i.n. immunization with MeV alone, the early activation of B cells in MDLN, spleen and bone marrow was confirmed at day 12. At day 35, B cell numbers in MDLN were low, high in bone marrow and higher in spleen, and neutralizing antibodies could be measured. When i.n. immunization with MeV was compared to immunization with MeV plus the combination of pI:C/ODN 2216, a slight increase in B cell numbers was found on day 12 and 35, but no increase in neutralizing antibody titers at day 35. After immunization with MeV in the presence of inhibitory MeV-specific IgG, no neutralizing antibodies were induced and B cell numbers were as low as determined before (see Figure 4) after i.n. immunization. After immunization with MeV and the combination of pI:C/ODN 2216 B cell numbers were restored to levels as in animals immunized with MeV in the absence of inhibitory IgG, and neutralizing antibodies were detected on day 35.

Similarly, s.c. immunization with MeV led to early activation of B cells in spleen and bone marrow at day 12, with increased numbers in spleen and bone marrow, and neutralizing antibodies at day 35. After s.c. immunization with MeV and pI:C/ODN 2216, B cell numbers were comparable to MeV immunization alone but the titer of neutralizing antibodies was increased on day 35. After s.c. immunization with MeV in the presence of inhibitory MeV-specific IgG, no neutralizing antibodies were induced, and B cell numbers in spleen and bone marrow were similarly low as after i.n. immunization with MeV in the presence of inhibitory MeV-specific IgG. After immunization with MeV and pI:C/ODN 2216, B cell numbers in spleen and bone marrow were reduced at day 12 compared to immunization with MeV in the absence of inhibitory IgG. However, at day 35 B cell numbers and neutralizing antibodies were comparable to immunization with MeV alone. In summary, these data demonstrate that MeV with the combination of pI:C/ODN 2216 restores the B cell and antibody response in the presence of inhibitory antibodies after both i.n. and s.c. immunization.

### Type I interferon acts through both type I interferon receptor and CD21

**Figure 6:**
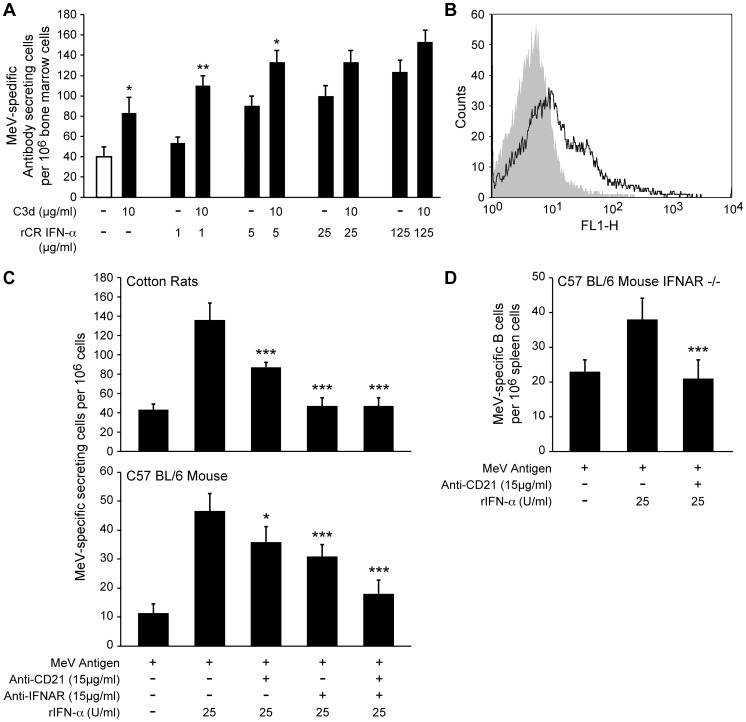
Interferon alpha uses CD21 as a functional receptor to support B cell activation. A) Bone marrow cells from MeV-immune cotton rats were stimulated with MeV antigen alone. After addition of C3d complement protein stimulation of B cells was increased. At lower concentrations, a synergistic stimulatory effect could be achieved by addition of interferon α. B) Cotton rat spleen cells were stimulated with LPS for 72 hours and stained with a cross-reactive serum specific for human/mouse/rat CD21 (black line, control as grey area). C) Spleen cells from MeV-immune cotton rats or C57 BL/6 mice were stimulated with MeV. Addition of cotton rat or mouse interferon α led to an increased B cell response. This stimulation was severely reduced after addition of antibodies specific for IFNAR or CD21. D) Spleen cells from MeV-immune C57 BL/6 mice with a deletion of the interferon receptor (IFNAR^−/−^) were stimulated with MeV alone, or mouse interferon α. The increase in stimulation was abrogated by blockade of CD21 by antibody. Each bar graph represents the mean ± SD of triplicate wells. Statistical analysis was done by ANOVA (* p<0.05, ** p<0.01, *** p<0.001). Every graph is representative for two or three experiments.

In this and other studies, interferon α proved to be a stronger stimulator of the B cell response than other cytokines including IL-6. A potential explanation might be provided by recent data which demonstrate that interferon α binds to CD21 with the same avidity as its natural ligand, C3d, which also stimulates B cell responses [30]. In consequence, IFN α is able to stimulate IFN-dependent genes through CD21 [31]. However, whether binding of IFN α to CD21 is also able to stimulate B cell responses has not been investigated. In order to determine whether the combined use of CD21 and IFN-R as receptors might be beneficial for B cell stimulation, we stimulated B cells with the natural ligand for CD21, C3d, and the natural ligand for IFN-R, IFN α, and a combination of both in an ELISPOT assay. C3d as well as IFN α were able to increase the B cell response in this assay system (Figure 6A). When a constant amount of C3d was combined with increasing amounts of IFN α, the stimulation was always stronger than if either one was used alone. However, with saturating amounts of IFN α, the synergistic effect decreased. These data demonstrate that the engagement of both CD21 and IFN-R stimulates B cells in a synergistic fashion. To find out whether B cells could also be stimulated by binding of IFN α to CD21, we stimulated B cells from MeV-immune cotton rats and mice with MeV and IFN α, and subsequently blocked CD21, IFN-R, or both by antibody (Figure 6C). Antibodies against both molecules led to a reduction in B cell stimulation through IFN α, indicating that CD21 can function as receptor for IFN α. To investigate whether CD21 alone could stimulate B cell responses in an IFN α dependent manner, we immunized mice with a deletion in the IFN-R gene with MeV. B cells from these mice could be stimulated with IFN α in vitro (Figure 6D). This effect was abolished by blockage of CD21 with antibody. These data demonstrate that CD21 can act as a receptor for IFN α and stimulate B cells, and it might explain the strong stimulatory effect IFN α has on B cells.

## Discussion

The inhibition of seroconversion after immunization in the presence of maternal antibodies is a well-documented phenomenon. The lack of generation of antibody suggests an impairment of B cell activation and development, although this was never proven. Our data demonstrate clearly that the inhibition in the generation of antibody correlates with the inhibition of B cell development, and particularly with early activation of B cells in draining lymph nodes. We have shown previously that the inhibitory action of maternal IgG is mediated through a complex of MeV vaccine/IgG cross-linking the B cell receptor (BCR) with the inhibitory receptor CD32 [26]. This inhibition could be partially overcome by MeV-specific IgM which crosslinks complement receptor 2/CD21 with BCR through a complex of MeV vaccine/IgM/complement protein C3d. It could also be partially overcome with viral vector systems which provided MeV hemagglutinin as antigen and induced type I interferon which is known to act through IFN-R [28]. This is consistent with data demonstrating that the induction of type I interferon supports B cell differentiation [29] and antibody secretion from B cells [32] [33] [34] [35]. Based on our data, the strong stimulatory activity of type I interferon on B cell responses seems to be due to the dual use of both CD21 and interferon receptor as functional receptors on B cells. The critical role of CD21 in the B cell response has been shown in Cr2−/− mice that are deficient in CR2 ( = CD21). Cr2−/− mice lack CD21 on both B cells and follicular dendritic cells [36]. They demonstrate substantial defects in antigen-specific, T cell-dependent and T cell-independent humoral immune responses [37], [38], [39]. In addition, defects in B cell memory [40], [41] and the development of the natural antibody repertoire [42], [43] are found in Cr2−/− mice. The natural ligand of CD21 is the complement protein C3d, and the binding of C3d to CD21 stimulates B cell responses. Interferon alpha contains a peptide sequence similar to one in C3d which is located at the C3d-CR2 binding site [30] and interferon alpha binds to CD21 with an affinity comparable to the natural ligand C3d. In vitro blockage of CD21 by antibody on human peripheral blood B cells diminishes the expression of interferon inducible genes [31]. In an ELISPOT assay, antibody secretion from B cells by type I interferon was clearly reduced when CD21 was blocked both on cotton rat and mouse B cells. Importantly, B cells from mice with a gene deletion in the interferon receptor respond to IFN α and this response can be blocked by antibodies against CD21. These data clearly demonstrate that CD21 is a functional receptor for IFN α on B cells. It seems likely that activation of the B cell through CD21 and IFN-R by type I interferon counteracts the inhibitory signal induced by CD32 through MeV-specific IgG and leads to a net stimulatory signal.

Previously, we have shown that the provision of type I interferon by immunization with NDV-H was able to partially restore the neutralizing antibody response in the presence of maternal antibodies. NDV interacts with TLR-7 and the RIG-I pathway which leads to the induction of type I interferon *in vivo* [44], [45]. TLRs use either the MyD88 (TLR-2, 4, 5, 7, 8, 9) or TRIF dependent signaling pathways (TLR-3, 4) (reviewed in [46]). It has been suggested that the expression of different TLR ligands by pathogens might enhance immune responses by signaling via both adapter pathways [47]. Here, we aimed to achieve the highest level of type I interferon by activating TLR-3 and TLR-9 through their agonists. We were able to show that a TLR-3 agonist (poly I:C) in combination with a TLR-9 agonist (ODN 2216) induced synergistically higher levels of type I interferon than either ligand alone. A combination of TLR agonists does not necessarily have a synergistic effect. Ghosh *et al* compared cytokine responses of all the possible combinations of known TLR ligands in human PBMCs [48]. TLR-9 agonist ODN 2216 produced high levels of interferon alpha but type I interferon induction through TLR-9 was downregulated in combination with TLR-7 or TLR-8 agonists. In contrast, TLR-3 induced significant amounts of type I interferon when used in a combination with agonists to TLR-2, 5, 7/8 [48] which support our data that co-stimulation of TLRs which are TRIF dependent (TLR-3) and MyD88 dependent (TLR-9) has a synergistic effect on type I interferon induction.

One possible application of our data would be the direct inoculation of IFN α with MeV vaccine in order to stimulate a better B cell and antibody response. In mice, the inoculation of a high dose of IFN α and influenza vaccine induced good T cell and B cell responses [49], but in humans this approach was not successful [50]. A possible reason is the difference in the effect of IFN α on the immune system of mice and humans. In humans, IFN α drives TH1 development and acts through STAT-4 which is not the case in mice [51]. The conclusion from these studies was that the mouse is not an informative animal model to study adjuvants which are targeted for use in the human respiratory system [50]. In cotton rats, inoculation of MeV vaccine and cotton rat IFN α intranasally did also not result in increased B cell responses (data not shown). The failure to stimulate the B cell response might be related to technical problems. Although doses comparable to the amounts found in lung lavage after TLR agonist application were used, it is possible that higher doses of IFN α are needed, or that pegylated interferon which is more stable in vivo is required. Alternatively, the cotton rat might be an animal model better suited to test intranasal adjuvants for humans [52], [53], [54]. This notion might be supported by the fact that in cotton rats a TLR-9 agonist optimized for human cells was most effective (ODN2216, data not shown), whereas in mice TLR-9 agonists optimized for mouse cells have to be used.

In summary, we have demonstrated that a combination of TLR-3 and TLR-9 agonists induces higher levels of type I interferon than either agonist alone. Subsequently, IFN α utilizes both IFN-R and CD21 as functional receptors for B cell stimulation and leads to restoration of B cell responses after immunization in the presence of inhibitory MeV-specific IgG.

## Materials and Methods

### Ethics statement

Animal use protocols for the experiments reported in this manuscript have been approved by the Institutional Animal Care and Use Committee of The Ohio State University in accordance with the Animal Welfare Act of the United States of America.

### Cotton rats

Inbred cotton rats (Sigmodon hispidus) were purchased from Harlan Laboratories, Inc. For immunization experiments in the presence of inhibitory antibody, MeV-specific IgG (NT of 100) was injected intraperitoneally (i.p.) into cotton rats to replace natural maternal IgG. One day later, animals were immunized with 10^5^ pfu of MeV (Schwarz strain) intranasally or subcutaneously with TLR agonists as indicated in each experiment. We have chosen human MeV-specific antibodies to standardize our experimental approach and because of experimental advantages: 1. the level of natural maternal antibodies in pups varies (presumably because of the suckling hierarchy), human antibodies metabolize faster (an experimental advantage) and can be distinguished by ELISA from cotton rat antibodies.

### Measles virus

The Schwarz vaccine strain of measles virus was grown and titrated on Vero cells as described [28].

### Antibodies

Human MEV-specific polyvalent IgG (Carimmune) with a neutralization titer (NT) of 640 was purchased from ZLB Behring. Neutralizing goat antibodies against cotton rat interferon alpha and IL-6 were purchased from R&D systems. Rabbit IgG specific for human/mouse/rat CD21 was purchased from Santa Cruz Biotechnology. Goat antibodies specific for human interferon receptor 1 and chicken antibodies specific for human interferon receptor 2 with neutralizing capacity were purchased from Thermo Scientific.

### Flow cytometry

Spleen cells were stimulated with 10 µg/ml LPS (Sigma) or 2.5 µg/ml Concanavalin A for 72 hours and lymphoblasts purified by centrifugation over a ficoll gradient. Subsequently, cells were stained with antibodies specific for CD21, or interferon receptor 1 or 2. The respective secondary antibody labeled with FITC was pre-absorbed with cotton rat serum for 1 hour on ice. Subsequently cells were analyzed by flow cytometry (Facscan, Becton Dickenson).

### TLR agonists

CpG ODN 2216 (5′-ggGGGACGATCGTCgggggg-3′ (bases shown in capital letters are phosphodiester, and in lower case phosphorothioate), type P ODN (CpG ODN 21798 [55]; 5′-tCGtCGaCGatCGgcgCGcgccg-3′) and type C ODN (CpG ODN M362, 5′-tcgtcgtcgttcgaacgacgttgat-3′) were purchased from Invivogen. Poly (cytidylic-inosinic) acid potassium salt (poly I:C) was purchased from Sigma.

### Bone marrow-derived plasmacytoid dendritic cell culture

Bone marrow derived dendritic cells were generated as described [28]. Bone marrow cells were cultured for 7 days in Advanced RPMI/5% FCS supplemented with 100 ng mouse Flt-3 ligand (R&D Systems). Every 2 days, fresh medium supplemented with Flt-3 ligand was added. On day 8, plasmacytoid dendritic cells were cultured with ODN 2216, poly I:C or measles virus, and 1 day later, the supernatants were harvested and analyzed by bioassay for the presence of type I interferon.

### Type I interferon bioassay

Samples from three wells were pooled and treated with 0.1M hydrochloric acid to inactivate IFN-γ for 2 hours and the acid was neutralized with sodium bicarbonate. Cotton rat osteosarcoma cells CCRT were incubated with serially diluted samples (in duplicate) or recombinant cotton rat IFN-α (R&D Systems) as standard for 24 hours. Subsequently, CCRT cells were infected with 10^3^ pfu of recombinant vesicular stomatitis virus expressing green fluorescent protein (rVSV-GFP). After 48 hours of incubation at 37°C, plates were evaluated for the presence or absence of green fluorescence on an Olympus 1 IX51 fluorescent microscope. IFN-α/β concentrations in samples were expressed as units with 1 unit being the equivalent of 1 pg of IFN-α. The threshold of detection was 16 units.

### Quantitative polymerase chain reaction to measure interleukin 6

Total RNA from cotton rat lung tissue was extracted using Qiagen RNeasy kit (Qiagen) and treated with DNase (Ambion). To generate cDNA, AffinityScript multiple temperature reverse transcriptase (Agilent technologies) and random primers (Promega) were used. Quantitative polymerase chain reaction was performed using a LightCycler RNA amplification kit with SYBR green I (Roche). The primer sequence for IL-6 was 5′-TTGGCACACTTAGGCACAGC-3′ (forward) and 5′-CAAAAGGACTGGCCGAGGAC-3′ (reverse), and the plasmid pCR-script Amp SK(+)-Cotton rat IL-6 [56] was used as standard. Data were analyzed using LightCycler software version 3. Quantification was based upon fit point analysis with arithmetic baseline adjustment. Melting peak and melting curve analyses were performed using the polynomial calculation method.

### Antibody neutralization assay

Cotton rat serum samples were two-fold serially diluted and incubated with 50 pfu MeV strain NSE for one hour at 37°C in a 96-well. One hour post-incubation 10^4^ Vero cells were added per well. Five days post-infection cytopathic effect (CPE) was determined microscopically. The titer was defined as the reciprocal of the last protective serum dilution, as calculated from duplicate measurements.

### B cell ELISPOT assay

For the B cell ELISPOT assay, mediastinal lymph node cells, bone marrow cells or spleens from measles virus immune cotton rats four to eight weeks after s.c. immunization with 10^5^ pfu of MeV (Schwarz strain) were used. 96 well plates (Millipore) were coated with gradient-purified, UV-inactivated MeV antigen in sodium carbonate buffer (pH 9.6) overnight at 4°C. Serially diluted lymphoid cells were plated and cultured at 37°C in an incubator. After overnight incubation, plates were washed with PBS/0.05% Tween 20. Plates were incubated with rabbit anti-cotton rat IgG (Virion Systems) and subsequently with goat anti-rabbit alkaline-phosphatase conjugated IgG (Zymed) in PBS/10% cotton rat serum. For development of spots, plates were washed three times with PBS and TMB-HK (high kinetic 3,3′,5,5′-tetramethylbenzidine, Moss, Inc.) substrate was added. Plates were incubated at room temperature for 5 minutes and spots were counted using an ELISPOT plate reader (CTL).

### Statistical analysis

Multiple comparison analysis was done by one-way analysis of variance (ANOVA) using Graph Pad InStat 3 for Windows (GraphPad Software). The number of asterisks denotes the level of statistical significance (*p<0.05; **p<0.01; ***p<0.001).
